# Investigation of the activity of phosphothioate and phosphothioate-LNA-modified oligonucleotides against HIV-1

**DOI:** 10.3389/fmed.2026.1719202

**Published:** 2026-02-02

**Authors:** Ludmila Gotfrid, Kirill Elfimov, Maria Gashnikova, Aleksey Totmenin, Aleksandr Agaphonov, Natalya Gashnikova

**Affiliations:** Department of Retroviruses, State Research Center of Virology and Biotechnology “Vector”, Novosibirsk, Russia

**Keywords:** antiretroviral activity of modified oligonucleotides, cellular internalization, HIV infection, LNA-modification, modified oligonucleotides, phosphothioate modification

## Abstract

This study investigated the antiretroviral efficacy, toxicity profile, and cellular uptake of phosphothioate (PS) and PS/LNA-modified oligonucleotides within an in vitro HIV infection model. Phosphothioate (PS) oligonucleotides, designed to bind conserved regions of the HIV-1 genome, were modified at the 3’ and/or 5’ ends with LNA nucleotides. The antiviral properties of oligonucleotides against HIV-1 subtype A6 were evaluated using human MT-4 cell cultures. The antiretroviral activity of LNA-oligonucleotides against HIV-1 has been established. Variations in the 50% inhibitory viral reproductive dose (IC50) values among the oligonucleotides were observed, depending upon both the target and the incorporated LNA modification. Optimal IC50 values (90 ± 10 nM) were achieved using a PS oligonucleotide lacking LNA modifications, which targeted the HIV-1 integrase-encoding genomic region. Optimal HIV inhibitory action among LNA constructs was observed in an oligonucleotide with a 5’-end LNA modification targeting the HIV integrase region (IC50 = 1.12 ± 0.03 μM). The introduction of LNA modifications to PS oligonucleotides failed to enhance antiviral activity, as demonstrated by IC50 values revealing significant in vitro HIV-1 inhibitory capacity. The internalization of oligonucleotides demonstrating optimal IC50 values was investigated via flow cytometry and imaging techniques. Antisense oligonucleotides with single PS modification showed better antiretroviral activity with lower IC50 value compared to PS/LNA-modified antisense oligonucleotides. The reason for this difference is the better internalization ability of PS-modified oligonucleotides. The characteristic features included low toxicity (maintaining >92% viable cells after 48 h of culture), high cytoplasmic membrane sorption capacity (approximately 12% FAM + cells after 48 h), high penetration efficiency (approximately 98% FAM + cells showing cytoplasmic signal), and elevated internalization and entropy ratios.

## 1 Introduction

The maximization of antiretroviral therapy (ART) treatment coverage for HIV-positive individuals has demonstrably enhanced both life expectancy and quality of life (1). Although ART effectively reduces HIV-1 replication in humans to undetectable levels, viral latency necessitates continuous lifelong treatment (2, 3). An unavoidable consequence of this treatment is the emergence and dissemination of drug-resistant strains. For example, resistance to dolutegravir, specifically primary resistance, has been reported to range from 3.9 to 8.6%, increasing to 19.6% in individuals with a history of ART (4). Another significant issue is the low rate of treatment adherence, especially within the youth population (5). Resistance and low adherence, both independently and synergistically, may accelerate disease progression and restrict subsequent ART options (6). Innovative therapeutic strategies could mitigate the adverse effects of ineffective treatment on disease progression in patients exhibiting primary resistance or poor adherence. Thus, the imperative is to research and develop drugs with new mechanisms of action against HIV.

A potential therapeutic avenue for HIV infection involves the application of therapeutic oligonucleotides, a class of molecules with established efficacy in treating both hereditary and infectious diseases (7–12). Antisense oligonucleotides continue to be investigated as a promising class of ARTs. Some of them have already been in the clinical trial phase. Illustrative examples include Gem92 (targeting the gag gene region), AR177 (a pol gene fragment encoding integrase), and GPs0193 (targeting the tat gene region) (13).

The mechanism of action of antisense oligonucleotides is based on blocking the translation of key viral proteins or degradation of target RNA and DNA molecules through activation of cellular nucleases such as RNase H (14), blocking the stereochemical interaction between HIV reverse transcriptase and genomic RNA (15), and inhibiting the process of reading viral mRNA matrix by the ribosome during translation (16).

**Figure 1:**
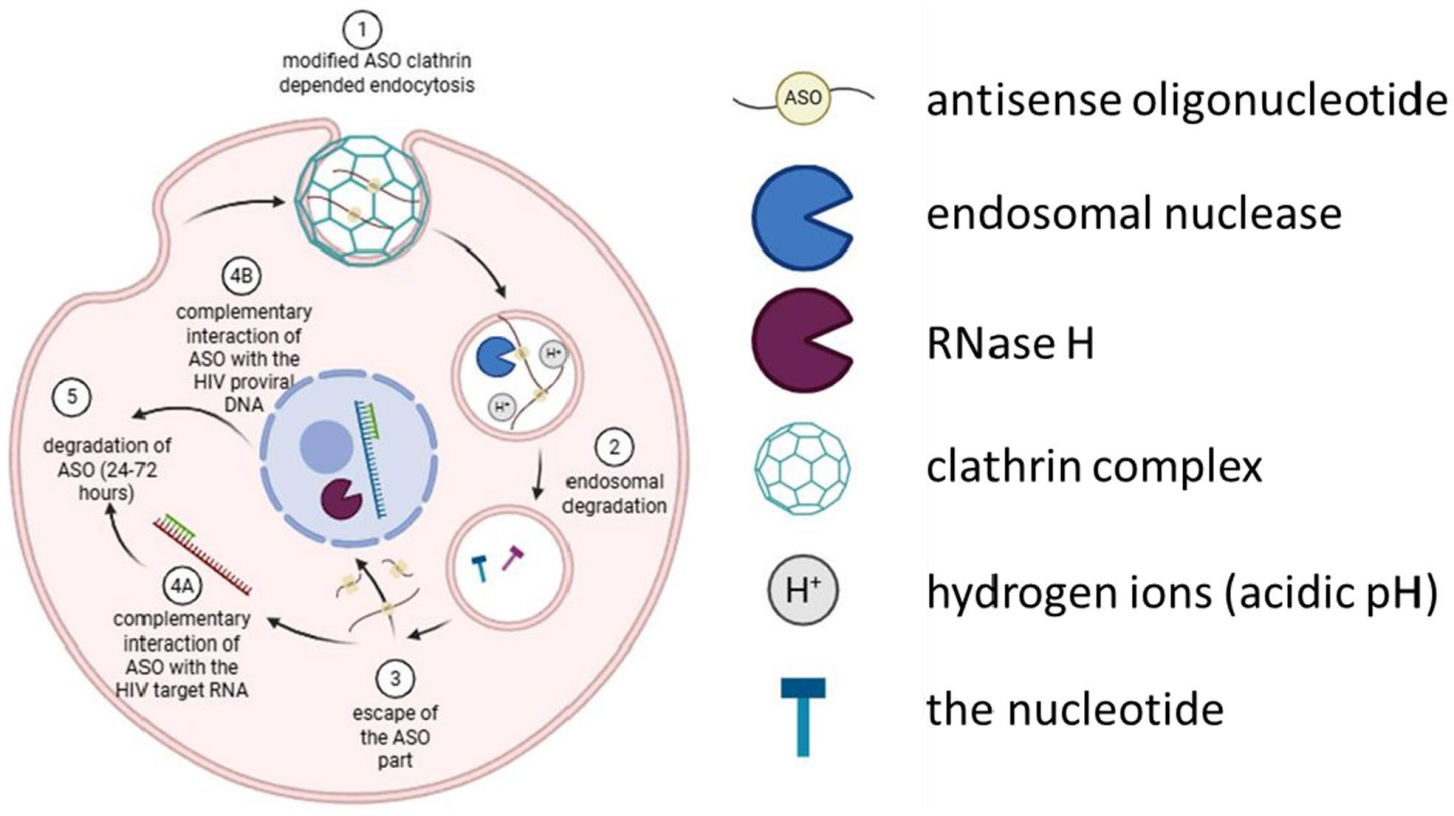
Membrane and intracellular transport of antisense oligonucleotides exhibiting thiophosphate and/or BNA/LNA modification. Created with BioRender.com.

The efficiency of antisense oligonucleotides is, in parallel, dependent on their ability to overcome various biological barriers, such as the cell membrane, endosomal retention, and nuclease activity (Figure 1) (17).

Oligonucleotide-target interaction efficiency is enhanced through the incorporation of several chemical modifications.

The antisense oligonucleotides (ASO) used were free of delivery vehicles, which can be considered an advantage. To enhance the internalization ability, the antisense oligonucleotides were chemically modified by adding phosphothioate (PS) and/or LNA (locked nucleic acid). It is known that such chemical modifications facilitate the penetration of oligonucleotides into cells without the use of transfection agents and other stimulation, which is extremely important when using ASOs as gene therapy agents (18, 19). Locked nucleic acid (LNA)-modifications are nucleotides in which the deoxyribose is modified by an additional bridge connecting a 2’-oxygen group to a carbon in the sugar moiety. The resulting fixed conformation leads to a higher melting point, thus enhancing resistance to denaturation and nuclease-mediated degradation (20). LNA-modification of oligonucleotides has been reported to increase the efficiency of inhibition of virus reproduction due to increased stability of DNA duplexes and increased affinity to target sequences (16, 21–25). The nuclear and cytoplasmic accumulation of LNA-oligonucleotides mediates the degradation of HIV RNA and DNA, which incorporate complementary antisense oligonucleotide target sequences.

This study aimed to comparatively assess cellular internalization and the *in vitro* ability of PS/LNA-modified or just PS oligonucleotides to inhibit HIV-1 replication. The antiviral properties of oligonucleotides were examined in human lymphoid cells infected with HIV-1 subtype A6, the prevalent strain in the Russian HIV epidemic (26). In addition, we evaluated the internalizationefficiency of different chemical modifications of oligonucleotides in a comparative aspect.

## 2 Materials and methods

### 2.1 Cell culture

This work used immortalized laboratory MT-4 cell culture representing T-cell leukemia cells sourced from the NIH-ARP program. The cultivation of MT-4 cells was conducted using RPMI-1640 medium (Servicebio, China; Cat. No. G4534-500ML) supplemented with 10% heat-inactivated FBS (Gibco, Waltham, Massachusetts, United States; Cat, No. A5209402), 2 mM L-glutamine (Gibco, Waltham, Massachusetts, USA; Cat, No. 25030081), and 20 μg/mL gentamicin (Gibco, Waltham, Massachusetts, United States; Cat, No. 15750078) in a sealed culture dish within a 37 °C, 5% CO2 incubator. The seed concentration was 350,000 cells per 1 mL of medium.

### 2.2 Viral material

The HIV-1 sub-subtype A6 strain, characterized by a rapid/high replication phenotype and significant cytopathic effect, resulted in >90% cell death by day 5. The virus was pre-inoculated on MT-4 cell culture. The infectious virus was harvested on day five of cultivation. HIV p24 concentration in viral fluids was quantified, and oligonucleotide activity was assessed using an ELISA method (HIV-1 p24-antigen-ELISA-BEST kit; Vector-Best, Russia), following the manufacturer’s protocol. Reed-Muench methodology (27) was used to determine the tissue culture infectious dose (TCID50) of the virus.

### 2.3 Study of antiviral activity

Immediately before the assay, oligonucleotides were serially diluted, starting at a concentration of 10 μM and progressing in threefold increments. Three independent oligonucleotide dilution series were added to a 96-well plate previously seeded with cells at a density of 70,000 cells per well. Oligonucleotides were incubated with MT-4 cells for 2 h before HIV-1 introduction followed by virus removal and replacement with fresh growth medium. Post-incubation, a constant dose of virus equivalent to 300 TCID50 was administered to the cell culture. The incubation with the virus was performed for 5 days in a CO2 incubator at 37 °C and 5% СО2. Upon completion of viral incubation with oligonucleotides, culture media samples were analyzed for p24 protein using enzyme-linked immunosorbent assay (ELISA). The study was conducted using the “prevention” (“pre-treatment”) method, which involves introducing oligonucleotides into the cells prior to virus addition and allows modeling the situation of pre-exposure prophylaxis (PrEP). Three independent experimental runs yielded results that were subsequently compiled and analyzed. The values presented in the article are expressed as the mean ± standard deviation.

### 2.4 Assessment of oligonucleotide toxicity (LD50) using the MTT assay

The assessment of cytotoxicity of the investigated oligonucleotide preparations using the MTT assay was performed in a 96-well plate. Each well of the plate received 40,000 MT-4 cells in 100 μL of complete RPMI-1640 culture medium (Servicebio, China; Cat. No. G4534-500ML) with 10% FBS (Gibco, Waltham, Massachusetts, USA; Cat, No. A5209402). Oligonucleotides were titrated with a three-fold dilution step, using three replicates for each oligonucleotide, which were added to the corresponding wells containing the cell culture. Incubation of MT-4 cells with the investigated oligonucleotide dilutions was carried out for 5 days in a CO2 incubator at 37 °C and 5% CO2.

Subsequently, 20 μL of MTT reagent solution was added to each well containing oligonucleotide samples and to the cell control wells. After an additional 2-h incubation in a thermostat, the culture medium was removed from the plate wells, and the resulting formazan precipitate was dissolved in isopropanol by shaking for 30 min. After dissolving the crystals in isopropanol, the intensity of the blue color was measured using a spectrophotometer Varioskan LUX (Thermo Fisher Scientific, Waltham, Massachusetts, USA; Cat, No. VL0L00D0) at two wavelengths (540 and 690 nm). Cell viability was assessed based on the intensity of their conversion of soluble MTT (3-(4,5-dimethylthiazol-2-yl)-2,5-diphenyltetrazolium bromide) into formazan crystals.

### 2.5 Staining for the detection of early apoptotic and necrotic events

The Annexin V-AF 488 Apoptosis Detection Kit with Propidium Iodide (Lumiprobe, Westminster, Maryland, USA; Cat. No. 21172) was used according to the manufacturer’s instructions to differentiate between cells in the stages of apoptosis and necrosis. Gating of the populations was performed as follows (Fig.): (I) Population of viable cells: absence of fluorescent signal in the FITC channel and the PI channel; (II) Population of cells in the early apoptosis stage: presence of fluorescent signal in the FITC channel, absence of fluorescent signal in the PI channel; (III) Cells that died by necrosis: absence of fluorescent signal in the FITC channel, presence of fluorescent signal in the PI channel; (IV) Cells that died by apoptosis: presence of fluorescent signal in both the FITC and PI channels.

### 2.6 Immunofluorescence intracellular staining for HIV-1 p55 protein

For staining of the HIV p55 protein in the cytoplasm of infected cells, the Anti-HIV1 p55 + p24 + p17 antibody (Abcam, United Kingdom; Cat. No. ab63917) was used according to the manufacturer’s recommendations; the fluorescent signal from the target was obtained using the secondary antibody Goat Anti-Rabbit IgG H&L (Alexa Fluor® 405) (Abcam, United Kingdom; Cat. No. ab175652), which was also used according to the manufacturer’s protocol. Cell fixation was performed using Fixation Buffer (BioLegend, San Diego, California, United States; Cat. No. 420801), and cell permeabilization was carried out with Intracellular Staining Perm Wash Buffer (10X) (BioLegend, San Diego, California, United States; Cat. No. 421002) used as a single wash. Cells were incubated with each antibody for 1 h in Cell Staining Buffer (BioLegend, San Diego, California, United States; Cat. No. 420801).

### 2.7 Imaging flow cytometry

The incubation of MT-4 cells with oligonucleotides was performed by adding the preparations to the nutrient medium to the final concentration. Incubation proceeded continuously throughout the experiment, with flow cytometry sampling performed at 1, 4, 12, 24, 36, and 48 h. The culture medium contained the indicated concentration of oligonucleotides throughout the incubation period. Cellular oligonucleotide uptake was assessed using flow cytometry on the Amnis FlowSight platform (Cytek® Biosciences, United States).

A 488 nm laser with a wavelength of 60 mW was used to excite the oligonucleotide-conjugated fluorescent tag FAM or AnnexinV-FITC, the viable PI dye, and light-field imaging (optical filters 532/55, 610/30, and 457/45, respectively). Images were acquired at a total magnification of 20 × (numerical lens aperture = 0.6) and a pixel size of 1 × 1 μm. A 488 nm laser with a wavelength of 60 mW was used to excite the oligonucleotide-conjugated fluorescent tag FAM, the viable PI dye, and light-field imaging (optical filters 532/55, 610/30, and 457/45, respectively). The Alexa Fluor 405 (AF405) dye was excited using a 405 nm laser with a power of 60 mW and a 457/45 filter. The images were captured at 20x magnification (numerical aperture, 0.6) and a pixel resolution of 1 μm x 1 μm.

Fluorescence intensity measurements, both intra- and extra-cytoplasmic, were facilitated by the creation of cytoplasmic and membrane masks. The cytoplasmic mask was generated from a light-field image using the Adaptive Erode parameter (M01, BF, 67). The membrane mask comprised the parameters of the complete object mask derived from the light-field imaging channel, excluding the cytoplasm mask [Object (M01, BF, Tight) And Not AdaptiveErode(M01, BF, 67)].

Several new parameters, based on pre-existing and user-specified masks, have been introduced in the IDEAS 6.2 software. An Internalization parameter derived from the “Internalization” algorithm facilitated the determination of the internalization coefficient (IC). The second custom parameter, Entropy_1, incorporated a base Shannon entropy parameter with a granularity of 1 within the cytoplasm mask.

## 3 Elisa

The measurement of HIV p24 protein concentration in the culture supernatant was performed using the HIV-1 p24-antigen-ELISA-BEST kit (VectorBest, Russia; Cat. No. 0134) according to the manufacturer’s instructions. Optical density was measured using an iMark™ Microplate Absorbance Reader (Bio-Rad Laboratories, Inc., Hercules, California, USA; Cat. No. 1681130) at a wavelength of 450 nm.

### 3.1 Statictical analysis

The normality of data distribution was evaluated using the Shapiro–Wilk test. The homogeneity of variance was evaluated using the Levene’s test. Quantitative data (most parameters) were summarized using the mean and standard deviation, while qualitative parameters (% viable cells) were described by the median and 95% confidence interval (95% CI).

The significance of variations between comparison groups was determined using ANOVA, with Fisher’s criterion (F-criterion) indicated. Differences between individual oligonucleotide modifications were analyzed using a one-way analysis of variance, with subsequent pairwise comparisons employing the Tukey honestly significant difference (HSD) test to adjust for multiple comparisons. The effect size was calculated using omega-squared (ω^2^), which denotes the proportion of trait variability accounted for by differences between modifications.

Within each modification, the influence of incubation time on the measured characteristics was evaluated using repeated-measures analysis of variance.

Pairwise comparisons of time points were conducted using paired t-tests, with the Benjamin-Hochberg correction applied to adjust for multiple comparisons. To determine the effect of time, a generalized eta-squared (η^2^G) analysis was conducted. This metric represents the proportion of trait variance attributable to the time factor while controlling for both intra-group and inter-individual replicate variation.

Kendell correlation studies were used to establish the relationship between imaging flow cytometryresults and IC50. Across all the comparisons, a *p*-value of less than 0.05 indicated statistical significance.

Statistical analysis was performed using the R programming language (v. 4.3.0, R Development Core Team, 2012; Vienna, Austria). The data were visualized using GraphPad Prism software version 10.0.0 (GraphPad Software, Boston, Massachusetts, United States).

## 4 Results

### 4.1 Oligonucleotide design

**Figure 2:**
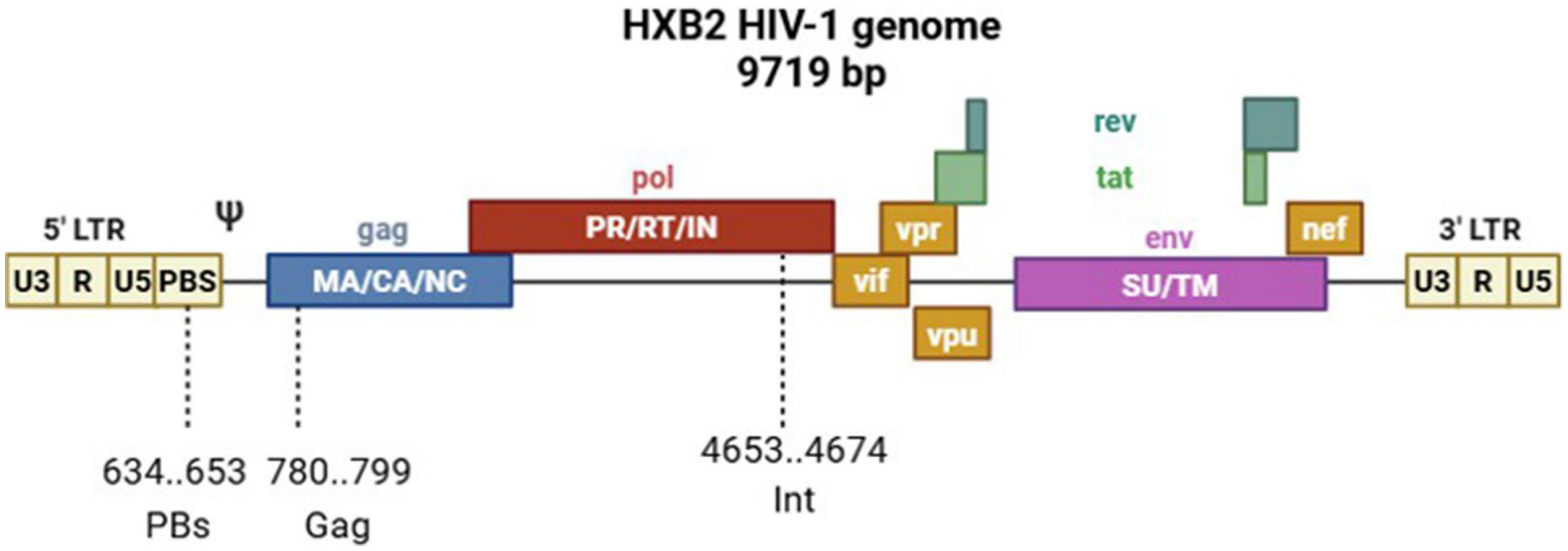
Scheme of the HIV-1 HXB2 genome showing custom oligonucleotide target sites.

For oligonucleotide targeting, we selected highly conserved sequences in the HIV-1 genome that are vital for the virus replication. These sequences are located in the primer-binding site region (PbS, involved in cDNA synthesis during reverse transcription), in the *pol* gene (Int, a gene fragment encoding a virus integrase that is responsible for integrating proviral DNA into the host cell genome), and in the HIV-1 *gag* gene (Gag, encoding capsid proteins; Figure 2).

To ensure potent and broad-spectrum antiviral activity, we selected target sequences within three conserved genomic regions of HIV-1 that are vital for viral replication: the primer-binding site (PBS), the integrase-coding segment within the *pol* gene (Int), and the *gag* gene. The choice of these specific targets was driven by their functional indispensability and high sequence conservation among HIV-1 strains, thereby reducing the probability of resistance development.

Our earlier work examining the internalization of oligonucleotides, modified and unmodified, showed phosphorothioate modification to be advantageous, a conclusion consistent with independent research (28–30). Additionally, oligonucleotides containing LNA at both ends of the sequence have been previously shown to function as potent inhibitors of HIV-1 expression in cell culture (16), and this modification has been suggested to be highly promising (31).

This study evaluated the antiviral properties of phosphorothioate-modified (PS) oligonucleotides, incorporating locked nucleic acid (LNA) modifications at either or both the 3’ and 5’ ends. PS oligonucleotides similar to the target oligonucleotides without the LNA modification were used as controls. Table 1 presents a summary of the oligonucleotide derivative structures utilized in this study. Each compound underwent an evaluation of its antiretroviral activity, determining the half-maximal inhibitory concentration (IC50).

**Table 1:** Sequences of oligonucleotides investigated in this work.

Oligonucleotide	Sequence (5’–3’)
Oligonucleotide derivatives targeting a conserved region of the HIV-1 genome in the primer-binding site region	
PbS	G^S^T^S^C^S^C^S^C^S^T^S^G^S^T^S^T^S^C^S^G^S^G^S^G^S^C^S^G^S^C^S^C^S^A^S^C^S^T
PbS_3’-LNA	G^S^T^S^C^S^C^S^C^S^T^S^G^S^T^S^T^S^C^S^G^S^G^S^G^S^C^S^G^L^C^L^C^L^A^L^C^L^T
PbS_5’-LNA	^L^G^L^T^L^C^L^C^L^CT^S^G^S^T^S^T^S^C^S^G^S^G^S^G^S^C^S^G^S^C^S^C^S^A^S^C^S^T
PbS_5’/3’-LNA	^L^G^L^T^L^C^L^C^L^CT^S^G^S^T^S^T^S^C^S^G^S^G^S^G^S^C^S^G^L^C^L^C^L^A^L^C^L^T
Oligonucleotide derivatives targeting a conserved region encoding HIV-1 integrase (pol gene)	
Int	C^S^T^S^T^S^G^S^A^S^C^S^T^S^T^S^T^S^G^S^G^S^G^S^G^S^A^S^T^S^T^S^G^S^T^S^A^S^G^S^G^S^G
Int_3’-LNA	C^S^T^S^T^S^G^S^A^S^C^S^T^S^T^S^T^S^G^S^G^S^G^S^G^S^A^S^T^S^T^S^G^L^T^L^A^L^G^L^G^L^G
Int_5’-LNA	^L^C^L^T^L^T^L^G^L^AC^S^T^S^T^S^T^S^G^S^G^S^G^S^G^S^A^S^T^S^T^S^G^S^T^S^A^S^G^S^G^S^G
Int_5’/3’-LNA	^L^C^L^T^L^T^L^G^L^AC^S^T^S^T^S^T^S^G^S^G^S^G^S^G^S^A^S^T^S^T^S^G^L^T^L^A^L^G^L^G^L^G
Oligonucleotide derivatives targeting a conserved region of the HIV-1 gag gene	
Gag	T^S^C^S^G^S^C^S^A^S^C^S^C^S^C^S^A^S^T^S^C^S^T^S^C^S^T^S^C^S^T^S^C^S^C^S^T^S^T
Gag_3’-LNA	T^S^C^S^G^S^C^S^A^S^C^S^C^S^C^S^A^S^T^S^C^S^T^S^C^S^T^S^C^L^T^L^C^L^C^L^T^L^T
Gag_5’-LNA	^L^T^L^C^L^G^L^C^L^AC^S^C^S^C^S^A^S^T^S^C^S^T^S^C^S^T^S^C^S^T^S^C^S^C^S^T^S^T
Gag_5’/3’-LNA	^L^T^L^C^L^G^L^C^L^AC^S^C^S^C^S^A^S^T^S^C^S^T^S^C^S^T^S^C^L^T^L^C^L^C^L^T^L^T
Negative control is not specific to the HIV-1 genome	
N_k	A^S^G^S^T^S^C^S^T^S^C^S^G^S^A^S^C^S^T^S^T^S^G^S^C^S^T^S^A^S^C^S^C
Oligonucleotide derivatives used for the investigation of cellular internalization capacity	
FAM-Int	[FAM]- C^S^T^S^T^S^G^S^A^S^C^S^T^S^T^S^T^S^G^S^G^S^G^S^G^S^A^S^T^S^T^S^G^S^T^S^A^S^G^S^G^S^G
FAM-Int_3’-LNA	[FAM]- C^S^T^S^T^S^G^S^A^S^C^S^T^S^T^S^T^S^G^S^G^S^G^S^G^S^A^S^T^S^T^S^G^L^T^L^A^L^G^L^G^L^G
FAM-Int_5’-LNA	[FAM]- C^L^T^L^T^L^G^L^A^L^C^S^T^S^T^S^T^S^G^S^G^S^G^S^G^S^A^S^T^S^T^S^G^S^T^S^A^S^G^S^G^S^G
FAM-Int_5’/3’-LNA	[FAM]- C^L^T^L^T^L^G^L^AC^S^T^S^T^S^T^S^G^S^G^S^G^S^G^S^A^S^T^S^T^S^G^L^T^L^A^L^G^L^G^L^G

S indicates the PS modification; L indicates the LNA modification; [FAM] indicates the fluorochrome tag, 6-carboxyfluorescein dye residue.

### 4.2 Study of antiretroviral activity

The antiviral efficacy of the oligonucleotides against HIV-1 strain subtype A6 was assessed using MT-4 cell suspension cultures, as detailed in the methodology section. Table 2 summarizes the specific oligonucleotide concentration at which there is a 50% suppression of HIV-1 reproduction in MT-4 cells.

**Table 2:** Data on the 50% concentration (IC50) determined for oligonucleotides inhibiting HIV-1 reproduction 5 days after infection of human cells with MT-4.

Oligonucleotide	IC50, μM	LD_50_, μM (MTT)
PbS	0.45 ± 0.05	>90
PbS_3’-LNA	3.27 ± 0.14	
PbS_5’-LNA	3.37 ± 0.14	
PbS_5’/3’-LNA	13.98 ± 0.93	
Int	0.09 ± 0.01	75.5 ± 5.5
Int_3’-LNA	1.30 ± 0.03	
Int_5’-LNA	1.12 ± 0.03	
Int_5’/3’-LNA	11.75 ± 0.64	
Gag	0.15 ± 0.02	>90
Gag_3’-LNA	2.34 ± 0.07	
Gag_5’-LNA	2.11 ± 0.07	
Gag_5’/3’-LNA	15.80 ± 0.84	
N_k	1.27 ± 0.10	>90

The optimal IC50 values were achieved using a PS oligonucleotide targeting the HIV-1 *pol* gene region that encodes the viral integrase, with the IC50 determined to be 90 ± 1 nM.

The LNA-modified oligonucleotides were found to exhibit antiretroviral activity *in vitro* at micromolar concentrations. The discrepancies in the activity between the target oligonucleotides with identical modifications are likely attributable to variations in target accessibility within the viral genome. Our results corroborate the observations of Jakobsen et al. (16) and Takahashi et al. (29), demonstrating comparable antiviral oligonucleotide efficacy against analogous targets in conserved HIV-1 regions at micromolar concentrations (IC50).

PS-modified oligonucleotides can exhibit nonspecific antiviral activity due to their phosphorothioate modification, which enables inhibition of viral particle fusion with the cell membrane. To verify the antisense effect of the developed ASOs, an oligonucleotide with a mismatched sequence “N_k” was synthesized (Table 1). Its antiretroviral efficacy (IC50 = 1.27 ± 0.10 μM) was higher than that of PS/LNA-modified ASOs but lower than that of PS-modified ASOs (Table 2).

The data analysis demonstrated that the observed differences in IC50 values were specific to oligonucleotides featuring LNA modifications. Regardless of 3’- or 5’-terminal LNA modification placement within the nucleotide sequence, antiviral activity among the tested oligonucleotides remained largely unaffected, with unmodified PS oligonucleotides demonstrating the highest HIV suppression. An in-depth analysis of oligonucleotide cellular internalization was performed to determine the ability of the construct to enter cells.

### 4.3 Study of oligonucleotide internalization

When performing fluorescence analysis of cell populations, viable and necrotic cells were differentiated since damage to the cytoplasmic membrane in dead cells leads to nonspecific binding of fluorescent probes and may also be accompanied by an increased level of autofluorescence.

**Figure 3:**
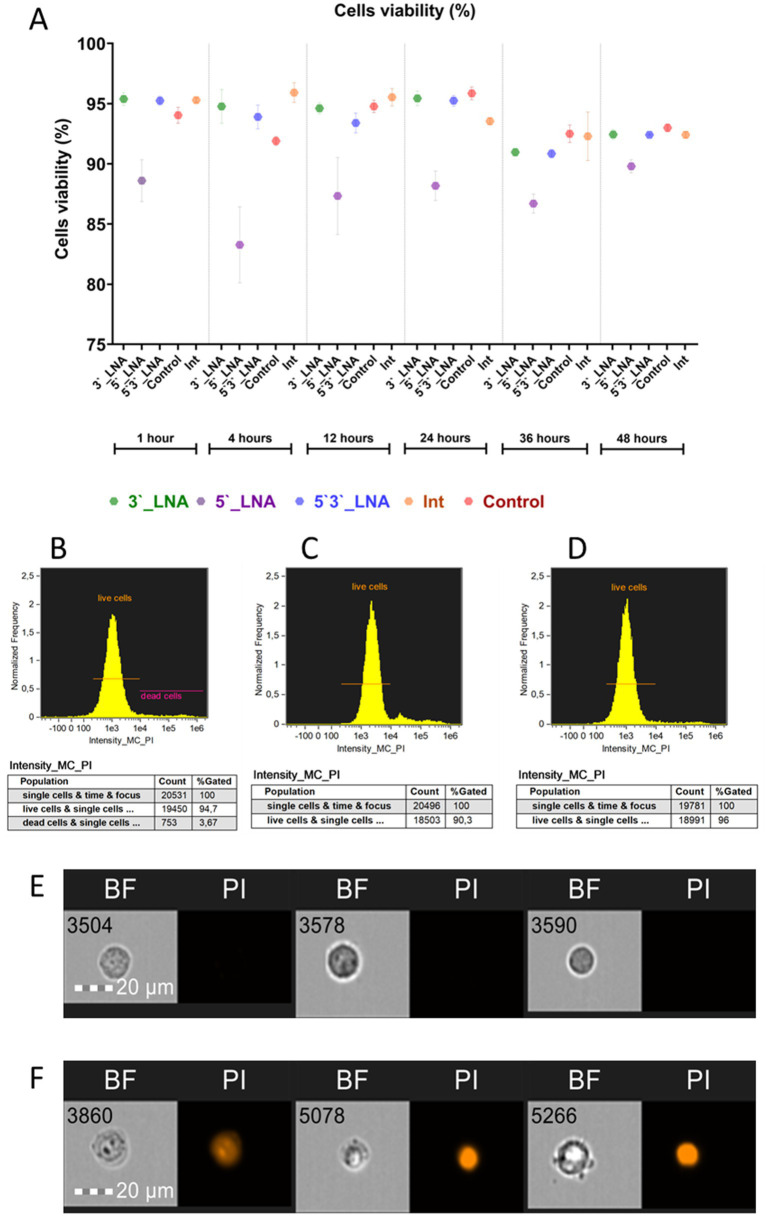
Cell viability of MT-4 culture cells upon the addition of oligonucleotides. **(A)** A graph showing cell viability as a function of added oligonucleotide modification or lack thereof (control) and time; **(B)** the population of live and dead cells in the control sample after 1 h of culturing; **(C)** the population of live cells in the sample containing 5’-LNA oligonucleotide; **(D)** the population of live cells in the sample containing the 3’-LNA oligonucleotide; **(E)** the images of live cells with no signal in the PI channel; **(F)** the images of dead cells with the fluorescent signal from PI. The images used in this analysis were obtained from MT4 fixed cells using imaging flow cytometry (Cytek® Amnis® Flow Sight). The cells were stained with ASO-FAM and PI to assess cell viability. Fluorescence was excited using a 488 nm laser with a power of 60 mW. The green fluorescence from FAM was detected in the second channel using a 532/55 nm filter, while the red fluorescence from PI was detected in the third channel using a 577/35 nm filter. The images were captured at a total magnification of 20 × (lens numerical aperture = 0.6), with a pixel size of 1 × 1 μm.

The issue mentioned above was addressed using propidium iodide, a viability marker with the property of penetrating only cells with compromised membranes to stain their DNA. Figure 3 illustrates the cell viability assessment results as a function of oligonucleotide modification.

In most of the samples containing modified oligonucleotides, cell viability values did not show statistically significant deviations from the control group during most of the experimental period. A notable exception was the 5’-LNA modification, which significantly impaired viability. Following a 60-min incubation period, viability was measured at 88.6 ± 1.74% (Figure 1C), subsequently declining to 83.27 ± 3.16% after 4 h (η^2^ = 0.84) and partially recovering to 89.8 ± 0.54% by 48 h (*p* = 0.338). No statistically significant variations (*p* > 0.05) were observed in the 5’-LNA-modified group, with viability decreasing steadily, unlike the control (*p* < 0.001) and other modified samples (*p* < 0.001 for all pairwise comparisons). The absence of full viability restoration by 48 h could indicate a potential effect of this modification on cellular metabolism.

Cell viability following single-end 3’-LNA modification exhibited no significant difference from the control. At the one-hour mark, viability measured 95.39 ± 0.53% (Figure 3D), declining to 92.44 ± 0.41% by 48 h. Although statistically significant temporal changes were observed (*p* = 0.004), the reduction in viability was not significantly greater than that of the control group (*p* = 0.393), suggesting a lack of specific cytotoxic effects from the 3’-LNA modification.

**Figure 4:**
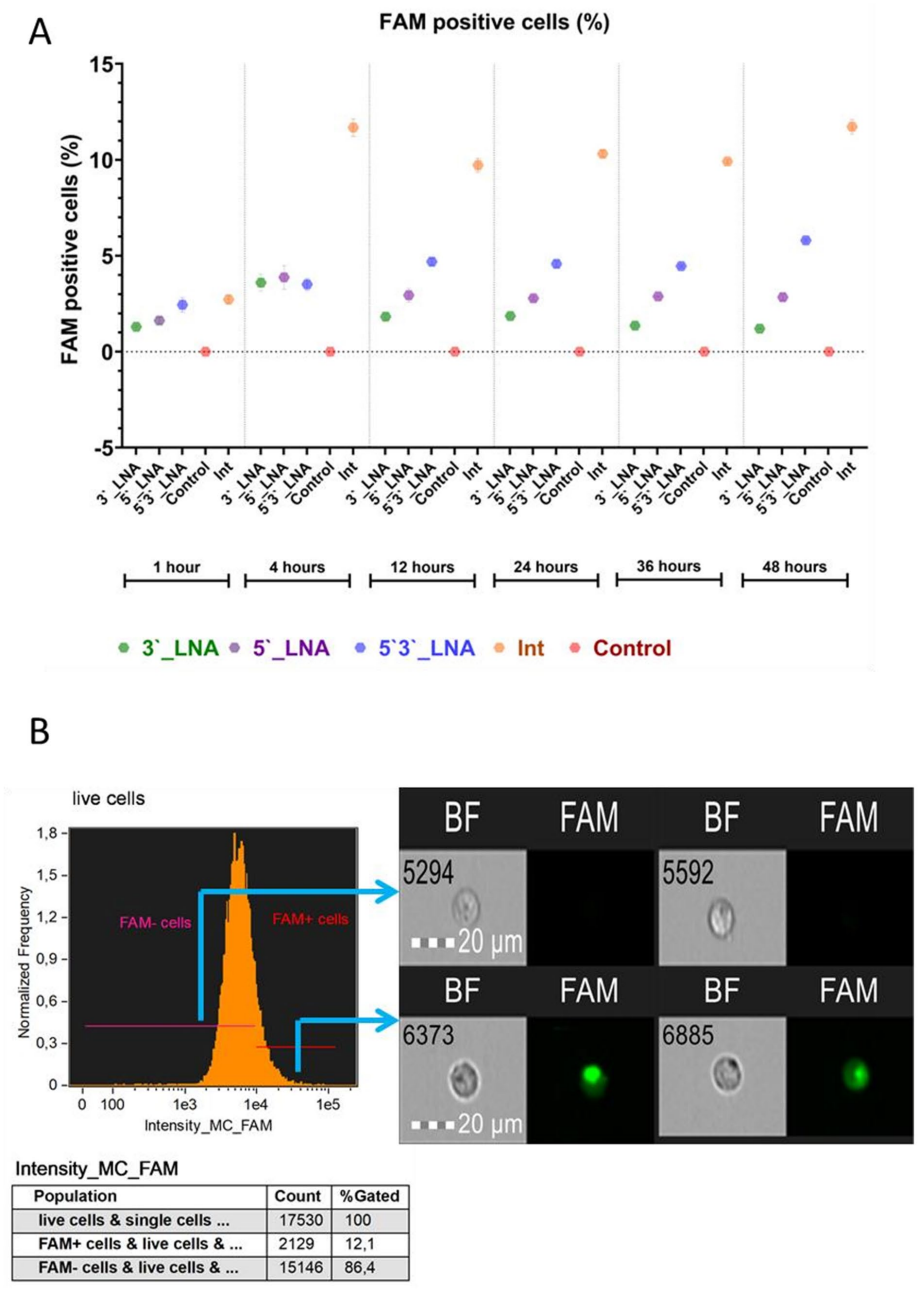
Absorption of antisense oligonucleotides by MT-4 cells: **(A)** A graph of the dependence of the number of viable cells on the used modification and cultivation time, with the median value indicated; tendrils show the error range of the mean; **(B)** The demonstration of the cells from populations with and without FAM + signal. The images used in this analysis were obtained from MT4 fixed cells using imaging flow cytometry (Cytek® Amnis® Flow Sight). The cells were stained with ASO-FAM and PI to assess cell viability. Fluorescence was excited using a 488 nm laser with a power of 60 mW. The green fluorescence from FAM was detected in the second channel using a 532/55 nm filter, while the red fluorescence from PI was detected in the third channel using a 577/35 nm filter. The images were captured at a total magnification of 20 × (lens numerical aperture = 0.6), with a pixel size of 1 × 1 μm.

**Figure 5:**
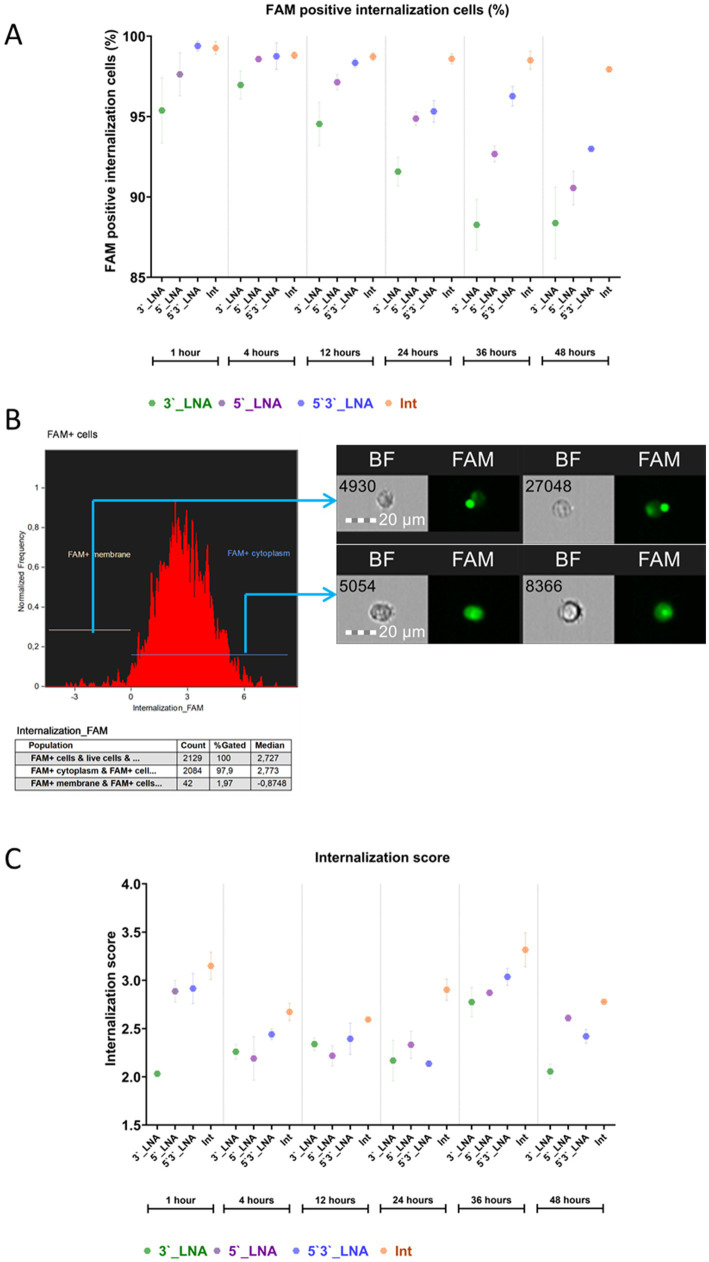
Evaluation of the penetration of antisense oligonucleotides of different modifications into the cytoplasm of MT-4 cells over time: **(A)** the fraction of cells with intracellular FAM signal; **(B)** visualization of cellular populations exhibiting FAM signal both at the cell membrane and within the cytoplasm. The images used in this analysis were obtained from MT4 fixed cells using imaging flow cytometry (Cytek® Amnis® Flow Sight). The cells were stained with ASO-FAM and PI to assess cell viability. Fluorescence was excited using a 488 nm laser with a power of 60 mW. The green fluorescence from FAM was detected in the second channel using a 532/55 nm filter, while the red fluorescence from PI was detected in the third channel using a 577/35 nm filter. The images were captured at a total magnification of 20 × (lens numerical aperture = 0.6), with a pixel size of 1 × 1 μm; **(C)** The value of the internalization coefficient depends on the modification used and the time.

The penetration study determined the percentage of cells that internalized oligonucleotides, both at the membrane and within the cytoplasm (Figure 4), and the proportion of events exhibiting cytoplasmic signal from labeled nucleotides (sample internalization, see Figure 5) (32, 33).

The experimental data obtained demonstrate the ability of all the chemical modifications of oligonucleotides under study to interact with the cell surface. However, their efficiency was found to be significantly different depending on the place of modification introduction. The statistical analysis using the F-criterion revealed significant differences between groups (*p* < 0.001), and the effect size (from ω^2^ = 0.95 to ω^2^ = 0.99) confirms that the chemical structure of oligonucleotides is a key factor determining their ability for cellular uptake. It is considered that the value of the criterion ω^2^ > 0.14 indicates a large influence of the studied trait on the observed differences (34). In this context, we observe a large effect of chemical modification of antisense oligo-nucleotides on the number of cells sorbing the FAM signal. Thus, the observed variations in the absorption efficiency of antisense oligonucleotides are almost entirely (95–99%) explained by the chemical modification used (PS-, 5’LNA, etc.) and not by other, including unaccounted factors.

The PS-modified sample (Int) exhibited the greatest absorption efficiency. Following a 1-h incubation period, no statistically significant difference in absorbance was observed between the unmodified oligonucleotide (Int) and the symmetrically 5’-3’ LNA-modified oligonucleotide (*p* = 0.53). However, statistically significant differences (*p* < 0.001) were detected when comparing the unmodified oligonucleotide (Int) with the terminally modified LNA variants (5’-LNA and 3’-LNA). During this period, the proportion of cells binding Int was 2.72 ± 0.243%, while the corresponding values for 5’3’-LNA, 3’-LNA, and 5’-LNA were 2.44 ± 0.374%, 1.3 ± 0.051%, and 1.62 ± 0.163%, respectively.

A four-hour incubation period revealed a statistically significant enhancement of the effects of thiophosphate modification (*p* < 0.001 for all comparisons). The observed hierarchy of uptake efficiency was Int > 5’3’LNA > 5’LNA > 3’LNA, indicating that the position of the LNA modification plays a critical role. The symmetrical arrangement (5’3’-LNA) may provide superior interaction stability within the cytoplasmic membrane and/or increased intracellular stability.

Nonetheless, the paramount metric for evaluating oligonucleotide efficacy is their capacity for cytoplasmic penetration, namely, internalization. Figure 5 presents the quantification of cytoplasm-localized, ligonucleotide-signaling cells.

The data obtained demonstrate that all the tested oligonucleotide modifications, phosphothioate (Int) and LNA modification (5’3’-LNA, 5’-LNA, 3’-LNA), efficiently penetrated into the cell cytoplasm, as evidenced by the proportion of FAM + cells exceeding 90% at the sampling time points. However, the statistical analysis revealed significant intergroup differences (*p* < 0.05), confirming the influence of chemical structure on uptake efficiency and intracellular stability.

Optimal results were observed with PS modification (Int), demonstrating FAM + cell proportions between 92.41 ± 0.252% and 95.53 ± 0.719% throughout the incubation period (1–24 h), with negligible variation between replicates. This observation suggests substantial resistance to degradation and stable interaction with cellular membranes.

No statistically significant difference (*p* > 0.05) in efficacy was observed between the symmetric LNA modification (5’3’-LNA) and Int within the first 12 h of cultivation. However, by 24 h, the difference reached statistical significance (*p* < 0.05), although the absolute difference was only 2–5%. A similar dynamics was observed for the LNA modification at the 5’-end. However, it should be taken into account that the detected statistically significant differences (2–5%) indicate the share of cells with intracellular localization of FAM in the group of FAM-positive cells. If we consider the differences in the proportion of cells with intracellular localization of FAM relative to all single cells, the differences are already 3–12%. This is especially true for the PS modification, which demonstrated values of 98–99% FAM + cells with signal internalization. The 3’-LNA modification, for example, had values of 86–89% of FAM + cells with internalization on the second day of cultivation.

The 3’-LNA modification exhibited the lowest efficiency, demonstrating a significantly reduced proportion of FAM + cells and increased inter-replicate variability. This may result from the expedited degradation of oligonucleotides within endosomes, a process mediated by exonucleases.

Oligonucleotide penetration efficiency in MT-4 cells was assessed using two parameters: the median internalization coefficient (IC), representing the intracellular oligonucleotide fraction relative to surface-bound molecules, and entropy, reflecting uniform intracellular distribution (Figure 4C). Statistical comparisons excluded control samples exhibiting a minimal FAM + cell population (≤0.003%).

The PS modification (Int) yielded maximal intercellular communication values at all cultivation phases. Within the initial 24-h period, no statistically significant differences (*p* > 0.05) were observed between Int, 5’-LNA, and 5’3’-LNA, with the exception of the 12-h mark. At this point, the IC for Int (2.59 ± 0.01) exhibited significantly higher values than that of 5’-LNA (2.22 ± 0.105; p 344 = 0.009). Within 48 h, the Int advantage had become clearly evident. The effect size (from ω^2^ = 0.6 to ω^2^ = 0.96) confirmed that it was the type of modification that caused the differences. Thus it can be concluded that the choice of chemical modification (PS-, LNA) has a major impact on the internalization ability of oligonucleotides, which was measured by counting cells with intracellular localization of oligonucleotide-bound FAM and internalization ratio.

A comparative study of LNA variants demonstrated a correlation between internalization and the location of modification. Following up to 36 h of cell culture, no statistically significant difference was observed between 3’ LNA and 5’ LNA or 5’/3’ LNA (*p* > 0.05). Nevertheless, a significant decrease in IC was observed by 48 h (*p* < 0.001), while 5’3’-LNA and 5’-LNA exhibited stable ratios of 2.42 ± 0.072 and 2.61 ± 0.039, respectively.

**Figure 6:**
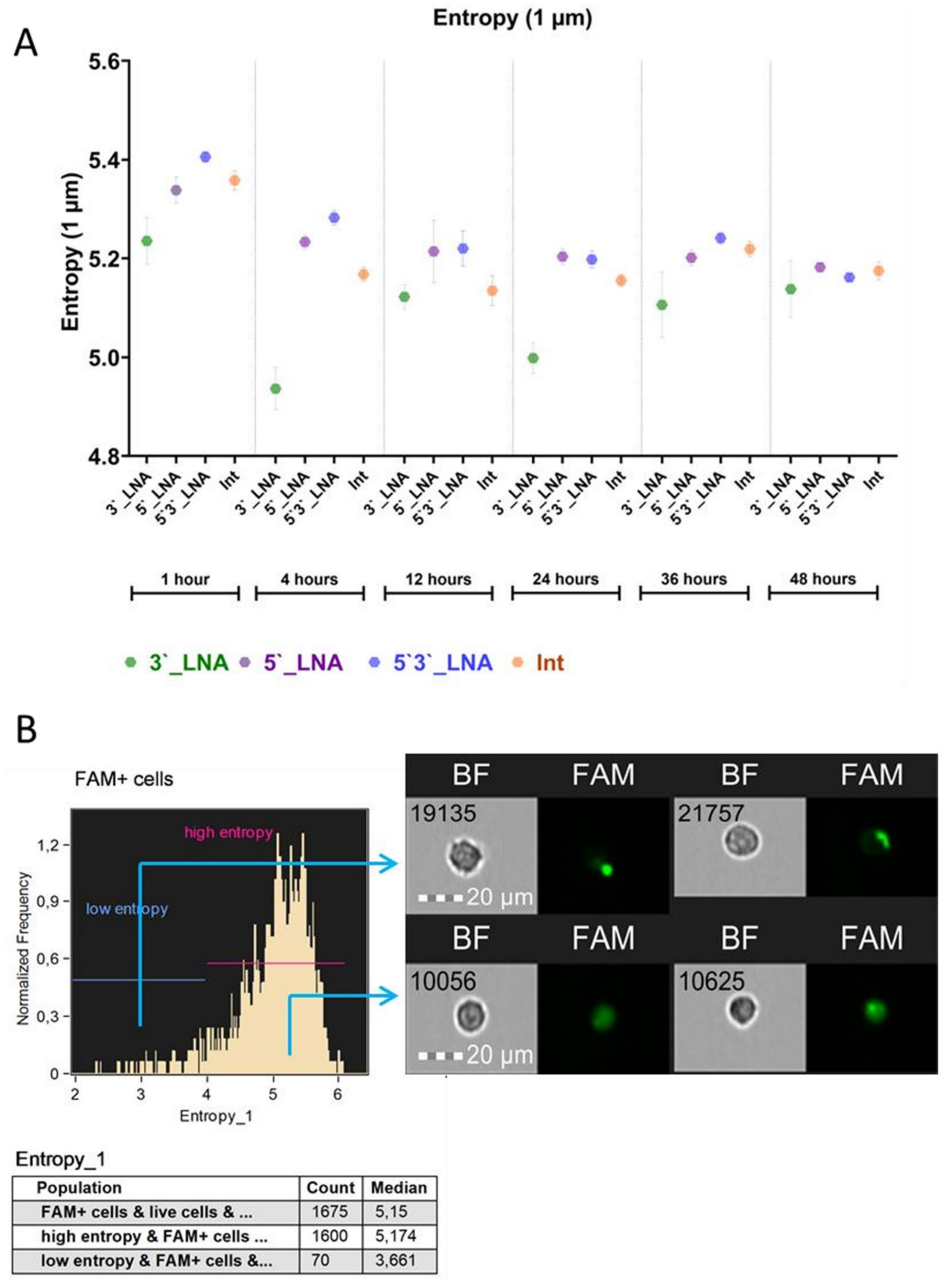
Results of the study of the entropy of antisense oligonucleotides in the cell cytoplasm: **(A)** Average entropy value with average error depending on modification and cultivation time; **(B)** Demonstration of the distribution of the FAM signal conjugated with oligonucleotides in the cytoplasm of cells with high and low entropy. The images used in this analysis were obtained from MT4 fixed cells using imaging flow cytometry (Cytek® Amnis® Flow Sight). The cells were stained with ASO-FAM and PI to assess cell viability. Fluorescence was excited using a 488 nm laser with a power of 60 mW. The green fluorescence from FAM was detected in the second channel using a 532/55 nm filter, while the red fluorescence from PI was detected in the third channel using a 577/35 nm filter. The images were captured at a total magnification of 20 × (lens numerical aperture = 0.6), with a pixel size of 1 × 1 μm.

Cellular uptake of oligonucleotides is a necessary, yet insufficient, prerequisite for functional efficacy. To bind to viral nucleic acids, oligonucleotides must evade degradation within endosomes, subsequently traversing the cytoplasm and karyoplasm to reach their primary targets (Figure 2). A key factor in their effectiveness is their ability to be uniformly distributed intracellularly, which determines their availability for interaction with target nucleic acids. The spatial distribution of compounds was quantified using the Shannon entropy parameter, derived from FAM fluorescence intensities within a composite mask encompassing both cytoplasmic and FAM fluorescence channel regions (Figure 6). The degree of molecular dispersal is reflected by entropy, with higher values signifying uniform diffusion and lower values indicating aggregation or localized accumulation.

The findings indicate a consistent cytoplasmic distribution of all oligonucleotides within the cells, with mean values being in the range of ≥5 throughout the cultivation period. The sample modified with 5’3’-LNA displayed peak entropy within the 1- to 36-h cultivation timeframe. Following a 48-h culture period, thiophosphate and 5’-LNA modification levels exceeded those observed in the sample with 5’3’-LNA modification (5.18 ± 0.008 and 5.16 ± 0.006, respectively). However, these discrepancies were not statistically significant (*p* = 0.948 and *p* = 0.841).

### 4.4 Investigation of the antiretroviral mechanism of ASOs

It is known that PS-modified oligonucleotides can exhibit antiretroviral activity by inhibiting the penetration/fusion of viral particles with the cytoplasmic membrane of the cell (35).

**Figure 7:**
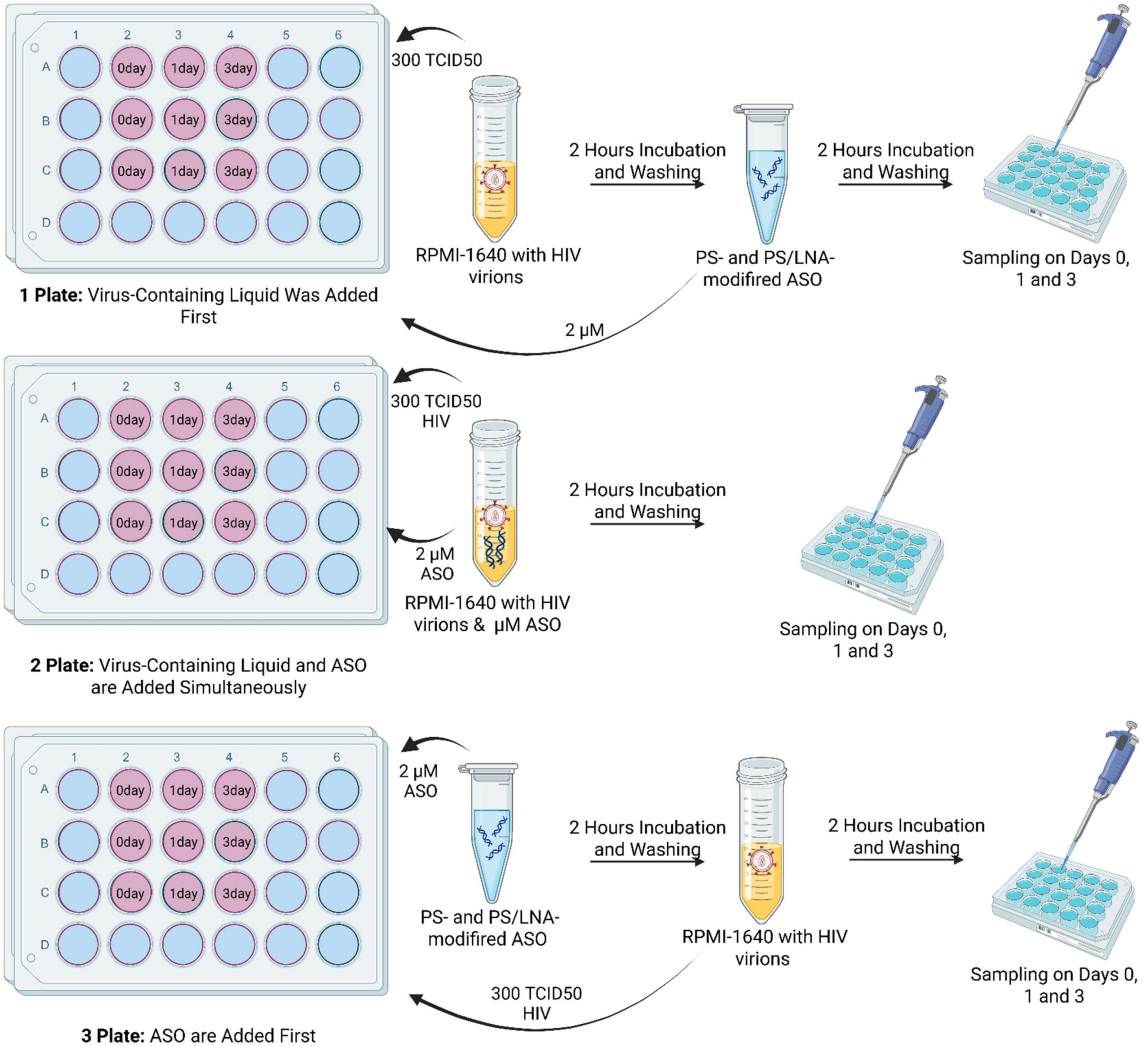
Schematic diagram of the experiment to determine the dominant mechanism of antiretroviral activity of the developed ASOs. Each ASO had its own plate for each method of addition: Plate 1 – viral material was added before ASOs; Plate 2 – viral material was added simultaneously with ASOs; Plate 3 – viral material was added after ASOs. Created with BioRender.com.

To clarify the mechanism of antiretroviral activity of the developed ASOs, an experiment was conducted involving different conditions for oligonucleotide addition: after incubation with viral material; simultaneous addition of viral material and ASOs; and addition of ASOs followed by infection (Figure 7). A control consisting of MT-4 cell culture with added viral material but without ASO addition (Viral Control) was used.

**Figure 8:**
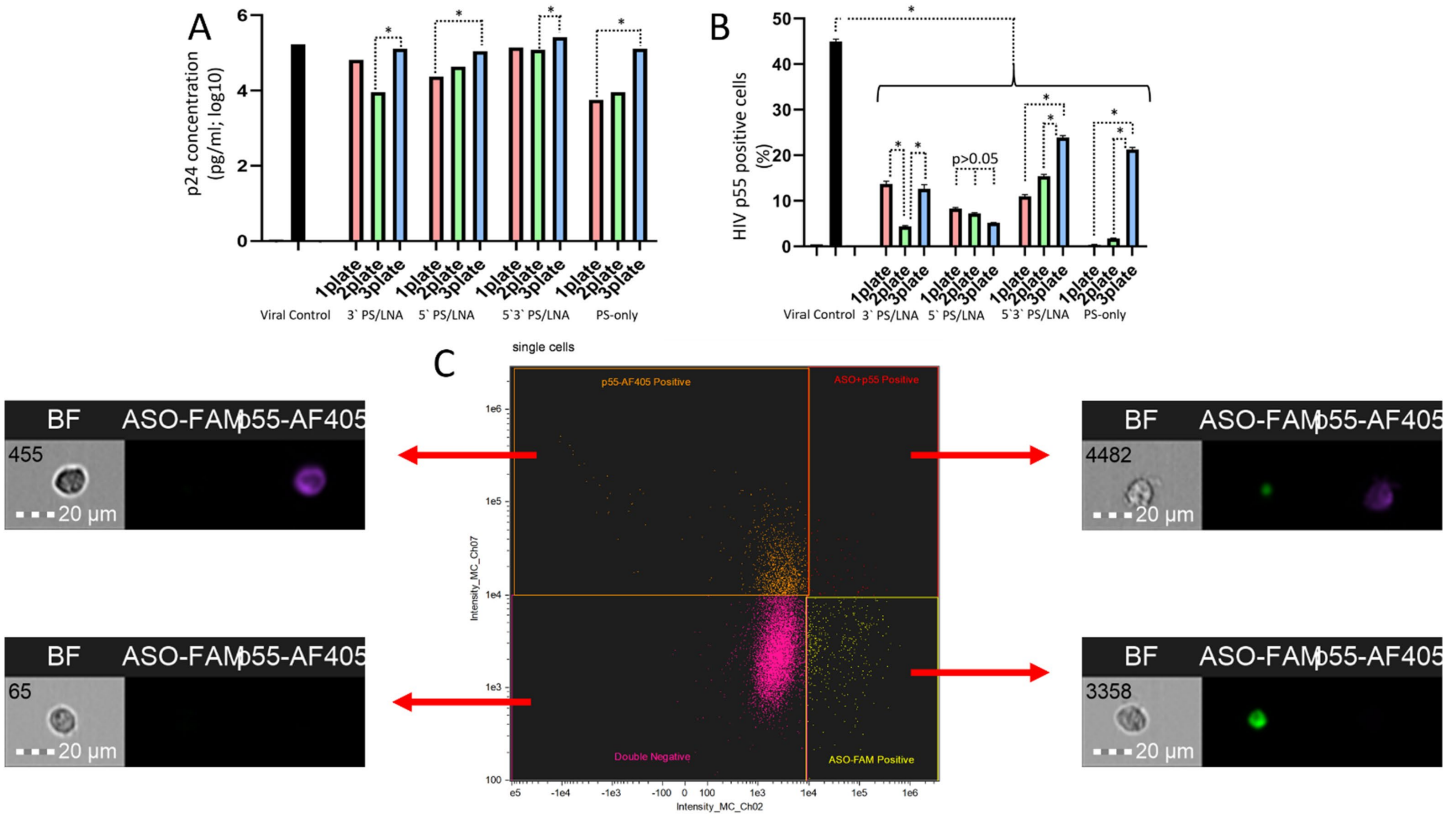
Differences in HIV replication inhibition depending on the ASO addition scheme (after incubation of cells with viral particles, during incubation of cells with viral particles, before incubation of cells with viral particles): **(A)** Amount of HIV p24 protein (log10) in the culture supernatant after 3 days of cultivation; **(B)** Number of cells containing the HIV structural protein p55 (precursor of p24) in the cytoplasm; **(C)** Gating strategy for the cell population during analysis of imaging flow cytometry results with examples of cells from different populations.

The measured parameters were the concentration of HIV p24 protein in the culture supernatant and the number of cells carrying the HIV p55 protein in the cytoplasm. The antisense effect of the ASOs would be more significant if the oligonucleotides were added after the viral particles (Plate 1). With this addition method, the ASOs could not exert the nonspecific PS-dependent effect caused by inhibition of viral particle fusion with cell membranes. The results show that the least effective method was the addition of ASOs before incubation with HIV virions (Figure 8).

The amount of HIV p24 protein in the culture supernatant on day 3 of cultivation was higher when ASOs were added to cells before incubation with viral particles (Figure 8A). The results of assessing the number of cells with intracellular localization of HIV p55 protein confirm this finding (Figure 8B), as in the samples with 3’LNA/PS, 5’3’LNA/PS, and PS-modified oligonucleotides, this specific method of oligonucleotide application demonstrated the lowest efficacy and a significantly greater number of p55-positive cells compared to the other addition methods (after incubation with viral particles; simultaneous incubation with viral particles).

Thus, the obtained data indicate a contribution of the antisense effect of the developed ASOs to the inhibition of HIV replication. However, the nonspecific action of the PS modification cannot be ruled out, as when using ASOs during the incubation of cells with viral particles, the antiretroviral effect was comparable. For instance, the amount of HIV p24 protein in the culture supernatant did not differ between Plate 1 and Plate 2 for all ASOs; in many cases, statistically significant differences between Plate 1 and Plate 2 were also not found when measuring p55-positive cells.

It is also worth noting that on day 3 of cultivation, p55-positive cells were detected in all samples. However, the population of cells with fluorescent signals from both ASO-FAM and p55-AF405 was the least abundant. This indicates that the p55 protein is practically undetectable in cells containing ASOs (Figure 8C). This finding further supports the presence of an antisense effect in the developed ASOs.

### 4.5 Assessment of ASO effects on cell viability, proliferation, and morphology

Additional experiments were conducted to more accurately assess the toxicity of the developed antisense oligonucleotides. These tests included the cultivation of MT-4 culture cells in the presence of antisense oligonucleotides at a concentration of 2 μM for 3 days. This experiment included an assessment of the number of proliferating cells, early apoptosis events, necrosis events, and some morphological characteristics (cell area, cell cytoplasmic membrane perimeter, cell aspect ratio, cell circularity, and cell nucleus area).

**Figure 9:**
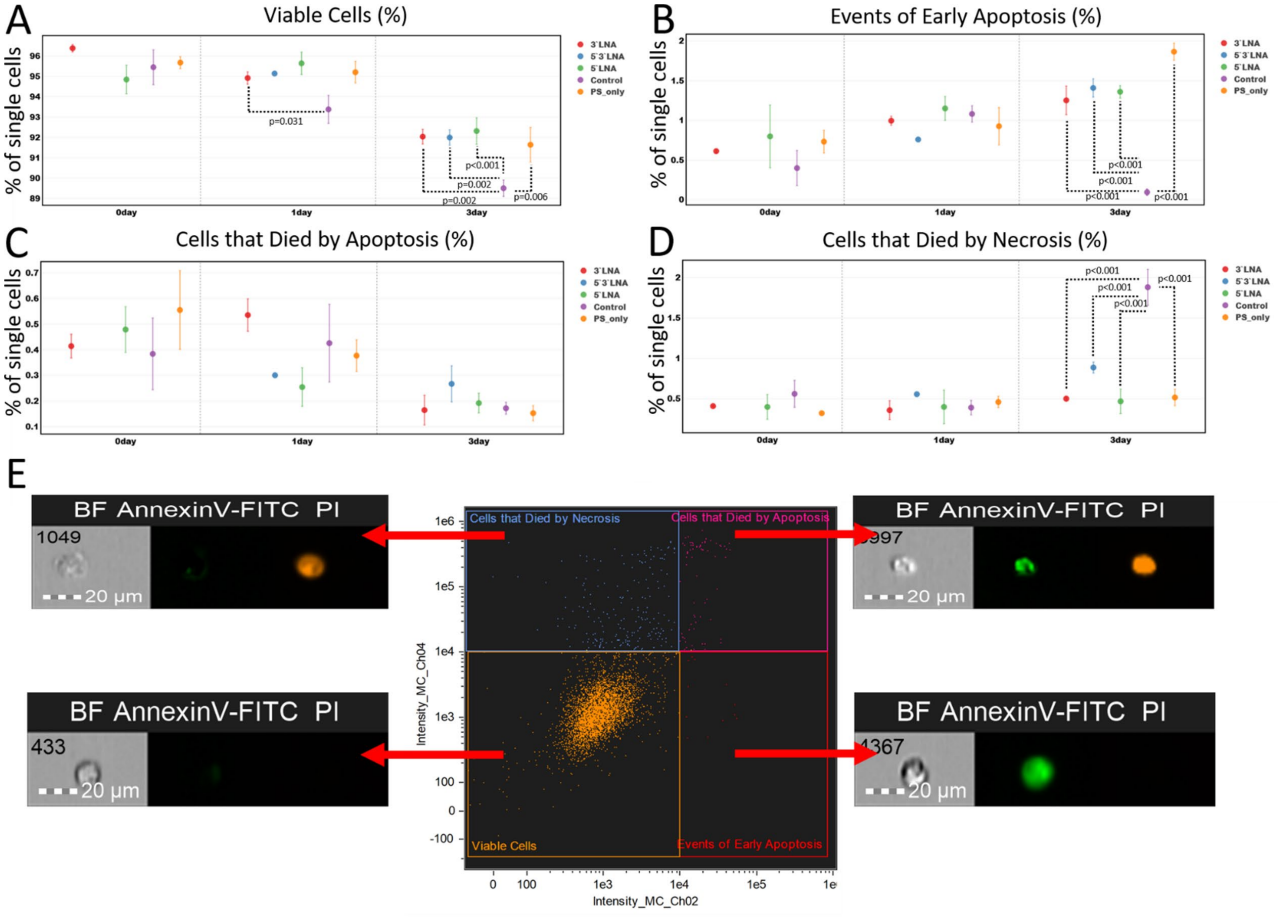
Results of the investigation of PS-modified and PS/LNA-modified antisense oligonucleotides against HIV in the context of their ability to initiate apoptosis/necrosis over three days (72 h) from the start of incubation: **(A)** Proportion of live single MT-4 culture cells during incubation with antisense oligonucleotides. The mean value and standard error of the mean are indicated; only statistically significant differences are marked, showing the *p*-value of the Tukey test; **(B)** Proportion of MT-4 culture cells in early apoptosis (AnnexinV-FITC+, PI- population) after incubation with antisense oligonucleotides. The mean value and standard error of the mean are indicated; only statistically significant differences are marked, showing the *p*-value of the Tukey test; **(C)** Proportion of MT-4 culture cells that died by apoptosis after incubation with antisense oligonucleotides. The mean value and standard error of the mean are indicated; only statistically significant differences are marked, showing the p-value of the Tukey test; **(D)** Proportion of MT-4 culture cells that died by necrosis after incubation with antisense oligonucleotides. The mean value and standard error of the mean are indicated; only statistically significant differences are marked, showing the *p*-value of the Tukey test; **(E)** Investigated cell populations after staining with AnnexinV-FITC and PI: double-negative cells are live cells; AnnexinV-FITC+ cells are in the stage of apoptosis; PI+ cells mark the population that died by necrosis; AnnexinV-FITC+ PI+ cells mark the population that died by apoptosis. Graphs were constructed using FluoSta v.1.0 software (41). Images were acquired using an Amnis FlowSight imaging flow cytometer (Cytek Biosciences, Fremont, CA, USA); fluorescence was excited using a 488 nm laser with a power of 60 mW. The green fluorescence from FITC was detected in the second channel using a 532/55 nm filter, while the orange fluorescence from PI was detected in the third channel using a 577/35 nm filter. The images were captured at a total magnification of 20 × (lens numerical aperture = 0.6), with a pixel size of 1 × 1 μm.

The results of the assessment of the ability of antisense oligonucleotides to induce apoptosis or necrosis showed that the investigated ASOs do not cause pronounced cytotoxicity (Figure 9).

Most of the statistically significant differences were found when comparing the number of live cells, the number of cells undergoing apoptosis, and the number of cells undergoing necrosis on the third day of the experiment. The first exception is the pairwise comparison of the number of live cells between the 3’PS/LNA-modified antisense oligonucleotide and the control sample after 1 day of the experiment (Figure 9A). However, this difference of ≈2% is not of high biological significance (3’LNA: 94.917% ± 0.300%; Control: 93.377% ± 0.689%). The second exception is the parameter of the number of cells that died by necrosis. This population did not demonstrate statistically significant differences between the study groups (Figure 9D).

On the third day of cultivation, the control group had the lowest number of viable cells, with statistically significant differences (*p* < 0.05) compared to all antisense oligonucleotides (Figure 9A). However, these differences (≈3%) may also lack high biological significance (3’LNA: 92.036% ± 0.359%; 5’LNA: 92.313% ± 0.647%; 5’3’LNA: 91.994% ± 0.361%; PS-modified oligonucleotide: 91.639% ± 0.851%; Control: 89.502% ± 0.402%).

It can be observed that on the 3rd day of the experiment, MT-4 cell culture samples containing antisense oligonucleotides had more cells undergoing apoptosis, with a difference within 2% (Figure 9B; 3’LNA: 1.253% ± 0.179%; 5’LNA: 1.361% ± 0.080%; 5’3’LNA: 1.410% ± 0.027%; PS-modified oligonucleotide: 1.866% ± 0.107%; Control: 0.097% ± 0.041%), but fewer cells undergoing necrosis with similar differences (Figure 9D; 3’LNA: 0.500% ± 0.029%; 5’LNA: 0.467% ± 0.152%; 5’3’LNA: 0.886% ± 0.107%; PS-modified oligonucleotide: 0.515% ± 0.101%; Control: 1.882% ± 0.222%).

The results of the cytotoxicity assessment under *in vitro* conditions confirm that the investigated antisense oligonucleotides do not lead to massive induction of apoptosis or cell death by necrosis. However, a small but statistically significant increase in the number of cells in the apoptosis stage for all antisense oligonucleotides on the 3rd day of the experiment raises concern, as the toxicity of the compounds under *in vivo* conditions may increase.

**Figure 10:**
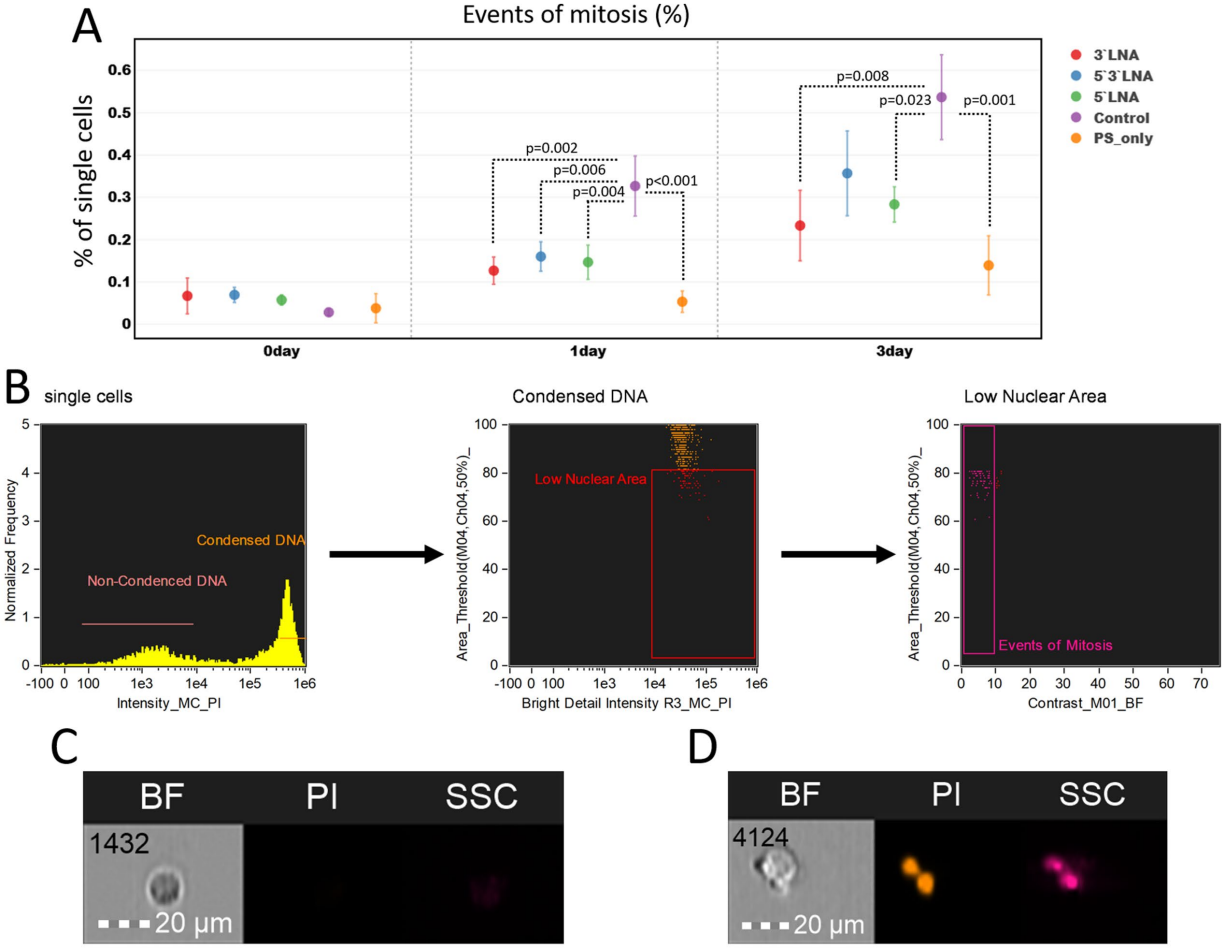
Investigation of proliferation inhibition caused by PS-modified and PS/LNA-modified antisense oligonucleotides with antiretroviral activity: **(A)** Proportion of dividing cells in MT-4 cell culture samples after a two-hour incubation with antisense oligonucleotides or without it (control). The mean value and standard error of the mean are indicated; only statistically significant differences are marked, showing the *p*-value of the Tukey test; **(B)** Gating strategy for MT-4 culture populations to determine the proportion of dividing cells; **(C)** Image of a cell in the G1 phase of the cell cycle (without DNA condensation); **(D)** Image of a cell undergoing mitosis. Graphs were constructed using FluoSta v.1.0 software (41). Images were acquired using an Amnis FlowSight imaging flow cytometer (Cytek Biosciences, Fremont, CA, USA); fluorescence was excited using a 488 nm laser with a power of 60 mW. The fluorescence from PI was detected in the third channel using a 577/35 nm filter. The images were captured at a total magnification of 20 × (lens numerical aperture = 0.6), with a pixel size of 1 × 1 μm.

We also performed an analysis of mitotic event counts to assess the effect of antisense oligonucleotides on cell proliferation ability (Figure 10).

At the start of the experiment, no significant differences in the number of proliferating cells were observed. After 24 and 72 h, these differences emerged, with the control sample (without ASO addition) showing the highest number of proliferating cells (24 h: 0.327% ± 0.071%; 72 h: 0.537% ± 0.100%), while all samples with added ASOs had a statistically significantly lower number compared to it. However, the biological significance of such differences requires further *in vivo* investigation, as the difference in the number of proliferating MT-4 culture cells in all cases was <0.5%.

**Figure 11:**
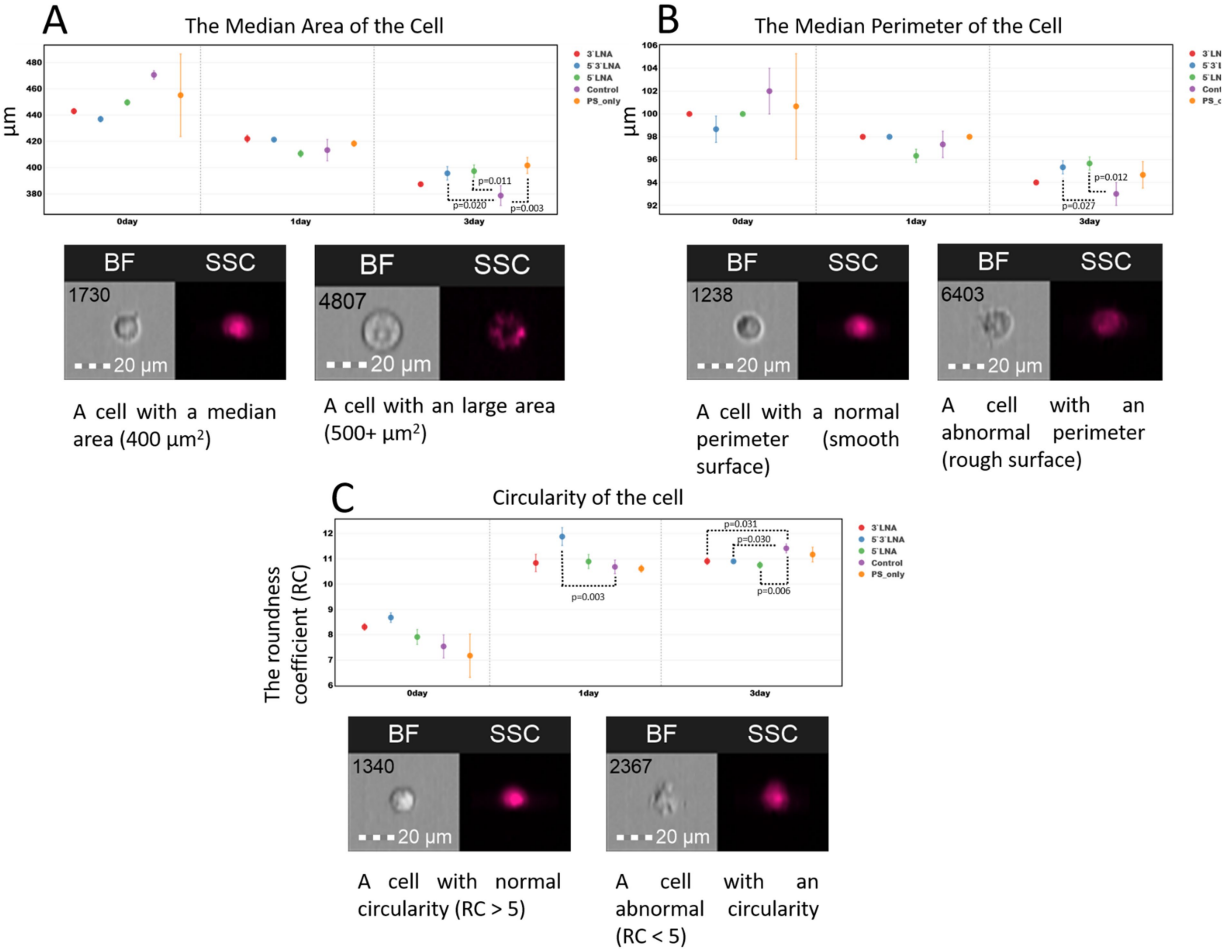
Morphological characteristics (area, perimeter, and circularity) of MT-4 culture cells without ASO addition and with the addition of ASOs with PS modification and PS/LNA modifications: **(A)** Median values of MT-4 cell area up to 72 h after a two-hour incubation with ASOs; **(B)** Median values of MT-4 cell perimeter up to 72 h after a two-hour incubation with ASOs; **(C)** Median values of the circularity coefficient of MT-4 cells up to 72 h after a two-hour incubation with ASOs. Graphs were constructed using FluoSta v.1.0 software (41). Images were acquired using an Amnis FlowSight imaging flow cytometer (Cytek Biosciences, Fremont, CA, USA); brightfield images were obtained using a 488 nm laser and a 457/45 light filter. The images were captured at a total magnification of 20 × (lens numerical aperture = 0.6), with a pixel size of 1 × 1 μm.

Several morphological cell parameters: cell area, perimeter, and circularity were measured as an additional assessment of ASO toxicity (Figure 11).

The results of the morphological measurements did not reveal significant changes in cell area and surface upon ASO addition (Figures 11A,B). Despite the presence of statistically significant differences, particularly after 72 h of cultivation, cell morphology remains normal following incubation with the investigated ASOs. We did not observe events with anomalous area (3,000 + μm^2^) or a large number of events with anomalous (shrunken, damaged) surface.

The measurement of the circularity ratio (RC) is based on the mean distance from the object’s center to its boundary, divided by the variation of this distance (to different points on the boundary). The higher the circularity coefficient, the more the cell shape approximates a perfect circle. The value recommended by Cytek Biosciences is RC ≈ 7. RC may vary within small limits (units) when using different cell cultures or experimental conditions; therefore, the recommended value is not an absolute boundary.

The results show that MT-4 culture cells do not exhibit biologically significant deviations from a rounded shape following a two-hour incubation with ASOs at a concentration of 2 μM (Figure 11C). The median RC value in all collected samples indicates that cells from the control sample maintain a rounded shape over 72 h (RC > 7). An exception was observed at the start of the experiment, where ASOs with a single PS modification (without LNA modification) showed a reduced RC value in one of the replicates. However, the differences compared to the control sample and other ASO samples did not demonstrate statistical significance.

The comprehensive cytotoxicity assessment, including the analysis of early apoptosis, necrosis, proliferation, and key morphological parameters, confirms the absence of pronounced cytotoxicity for the investigated ASOs. All constructs, regardless of the type of chemical modification, demonstrated minimal and biologically insignificant effects on the viability and morphology of the MT-4 cell line. Although statistically significant differences were observed, such as a slight increase in the proportion of apoptotic cells or a decrease in the number of dividing cells, they do not indicate pronounced nonspecific toxicity *in vitro*.

## 5 Discussion

This study assessed the antiretroviral efficacy and cellular uptake of phosphorothioate oligonucleotides incorporating LNA modifications at the 3’- and/or 5’-ends of the nucleotide sequence. The rationale for these modifications was based on their established properties: enhanced resistance to nucleolytic degradation owing to the substitution of oxygen by sulfur within the phosphodiester bond (16), improved target hybridization attributable to the conformational rigidity of LNA (36), and diminished negative charge, thereby promoting interaction with the cell membrane and endocytosis (30). The assessment of antiretroviral activity was conducted using infectious HIV-1 subtype A6 strains, with IC50 values determined. The most effective IC50 values were determined for a PS oligonucleotide directed against the integrase-coding sequence within the HIV-1 pol gene. This modification was previously studied as an inhibitor of HIV-1 gp41-mediated fusion and viral entry (35), yet a comprehensive evaluation of its cellular penetration was lacking. In an analogous study (37), the authors employed thiophosphate oligonucleotides containing imidazole and an amine group. Although several sequence variants exhibited activity, cytoplasmic uptake was not examined. The LNA modification, however, failed to improve oligonucleotide-mediated inhibition of HIV replication. The antiviral activity of oligonucleotides was most significantly reduced by incorporating LNAs at both ends of the sequence, compared to PS modification. This may reflect a reduced capacity for cellular uptake. Juliano et al. reported similar findings, attributing the reduced transmembrane permeability of excessively modified molecules to steric hindrance and compromised ligand function (30). Another study aimed at blocking HIV-1 gp120 also included work with different chemical modifications of antisense oligonucleotides. Interestingly, the authors demonstrated lower IC50 values for PS-oligonucleotides that were similar to our values. For example, the IC50 value of compound DBM-2198 was 0.08 ± 0.02 μM, while the best oligonucleotide targeting the integrase region and having only PS modification showed an IC50 value of 0.09 ± 0.01 μM (38).

Alternatively, some studies indicate a greater efficacy of LNA-modified oligonucleotides when compared to unmodified antisense oligonucleotides and DNAzymes. Those studies, however, utilized a plasmid vector that included portions of the HIV genome instead of an infectious, competent isolate (16). In addition, there is a gap in comparative penetration studies of PS- and PS/LNA-modified ASO. This study shows that the introduction of additional chemical modifications in the form of LNA can impair the anti-viral activity of ASO. This effect was shown to be at least partially related to the worse internalization ability of PS/LNA ASOs compared to ASOs carrying only PS modifications.

We investigated oligonucleotide cellular uptake via flow cytometry and imaging, quantifying absorption and penetration. Intracellular oligonucleotide distribution was characterized using the internalization coefficient (intracellular/membrane signal ratio) and entropy. Given the characteristics investigated, the PS modification (Int) proved to be the most effective, exhibiting low toxicity (cell viability >92% at 48 h), substantial cytoplasmic membrane sorption (≈12% FAM + cells at 48 h), significant penetration (≈98% FAM + cells with cytoplasmic signal), and high internalization and entropy ratios.

Typically, the IC50 value showed a positive correlation with oligonucleotide internalization efficiency. However, an exception was the 5’3’ PS/LNA-modified ASO, which exhibited better internalization compared to the 5’PS/LNA and 3’PS/LNA-modified ASOs, yet had a higher IC50 concentration. This may be due to difficulty in recruiting RNase H because of the lack of a “gap” of LNA-unmodified nucleotides in the sequence (39).

When considering the LNA modification group, it is worth noting the advantages of single-end modification at the 5’-end and at both ends of the molecule, with similar characteristics to the PS modification in terms of internalization and entropy up to 24 h of cultivation. However, they had fewer cells that absorbed oligonucleotides (3–6%) at the 48-h culturing time point compared with PS (≈12%). The 3’-LNA modification exhibited enhanced toxicity and markedly reduced absorption and cellular penetration. The observed effect is likely due to the complex interaction with the cell membrane and decreased susceptibility to exonucleases initiating degradation from the 5’ end of the DNA molecule. Similar works investigating the antiretroviral activity of LNA-modified oligonucleotides have generally utilized modification at both ends of the molecule (39, 40).

In conclusion, the question of the pronounced antiretroviral effect of ASOs despite the relatively small number of ASO-positive cells (≈10% by 48 h of cultivation) should be considered. This observation can be explained by two reasons: (I) the detection limit of the imaging flow cytometer, with a sensitivity threshold of approximately 10 MESF (Molecules of Equivalent Soluble Fluorochrome). Consequently, we may fail to detect ASO-positive cells containing small amounts of ASO, which nevertheless exhibit antiretroviral activity. (II) Another reason could be the nonspecific PS-dependent antiretroviral activity, which manifests in the extracellular environment as fusion inhibitors. Such oligonucleotides would exert their activity without entering the cells. The mechanism of action of the developed ASOs is based on the combined effect of the oligonucleotides as both fusion inhibitors and antisense sequences. The most pronounced nonspecific PS-dependent effect is observed for ASOs with single PS modification, which have a lower IC50 value compared to their LNA-modified analogs.

## 6 Conclusion

A comprehensive study of phosphorothioate oligonucleotides and PS oligonucleotides with additional introduction of LNA modification at the 3’- and/or 5’-ends of the nucleotide sequence in an *in vitro* model of HIV infection confirmed the ability of oligonucleotides at nanomolar concentrations to inhibit HIV-1 reproduction. This study demonstrated that LNA modifications in PS oligonucleotides did not enhance antiviral activity, providing a partial explanation for the observed intracellular oligonucleotide uptake patterns across various modifications.

All the oligonucleotides under study were found to display minimal toxicity and significant cytoplasmic membrane permeability in human lymphoid cells. Furthermore, the internalization factor of the fluorescent signal from all oligonucleotides was demonstrated to exceed 2, indicating substantial intracellular penetration beyond adsorption to the cytoplasmic membrane surface. The uniform distribution of oligonucleotides within the cell cytoplasm, as described, confirms their ability to diffuse freely within cellular compartments, exhibiting marked resistance to endosomal nucleases and a uniform distribution throughout the cell’s interior, improving the probability of encountering their target HIV-1 RNA.

The improved antiviral efficacy of phosphorothioate-modified oligonucleotides against HIV-1 is likely attributable to their advantageous characteristics, as demonstrated across most aspects of our investigation. We showed that PS/LNA modifications of antisense oligonucleotides were inferior to similar PS oligonucleotides in antiretroviral activity (IC50 0.09 ± 0.01 μM vs. 1.12 ± 0.03), which was attributed to the lower internalization capacity of PS/LNA oligonucleotides.

Our comparative analysis of modified oligonucleotide sets indicates that phosphorothioate oligonucleotides represent a highly promising approach to HIV-1 therapy, characterized by efficient cellular internalization and resistance to intracellular degradation.

## Data Availability

The raw data supporting the conclusions of this article will be made available by the authors, without undue reservation.
